# Supplementary figures and images for: Smart thermosensitive poloxamer hydrogels loaded with Nr-CWs for the treatment of diabetic wounds
Source: PLoS One. 2022 Dec 30;17(12):e0279727. doi: 10.1371/journal.pone.0279727 (PMC9803202; doi:10.1371/journal.pone.0279727)

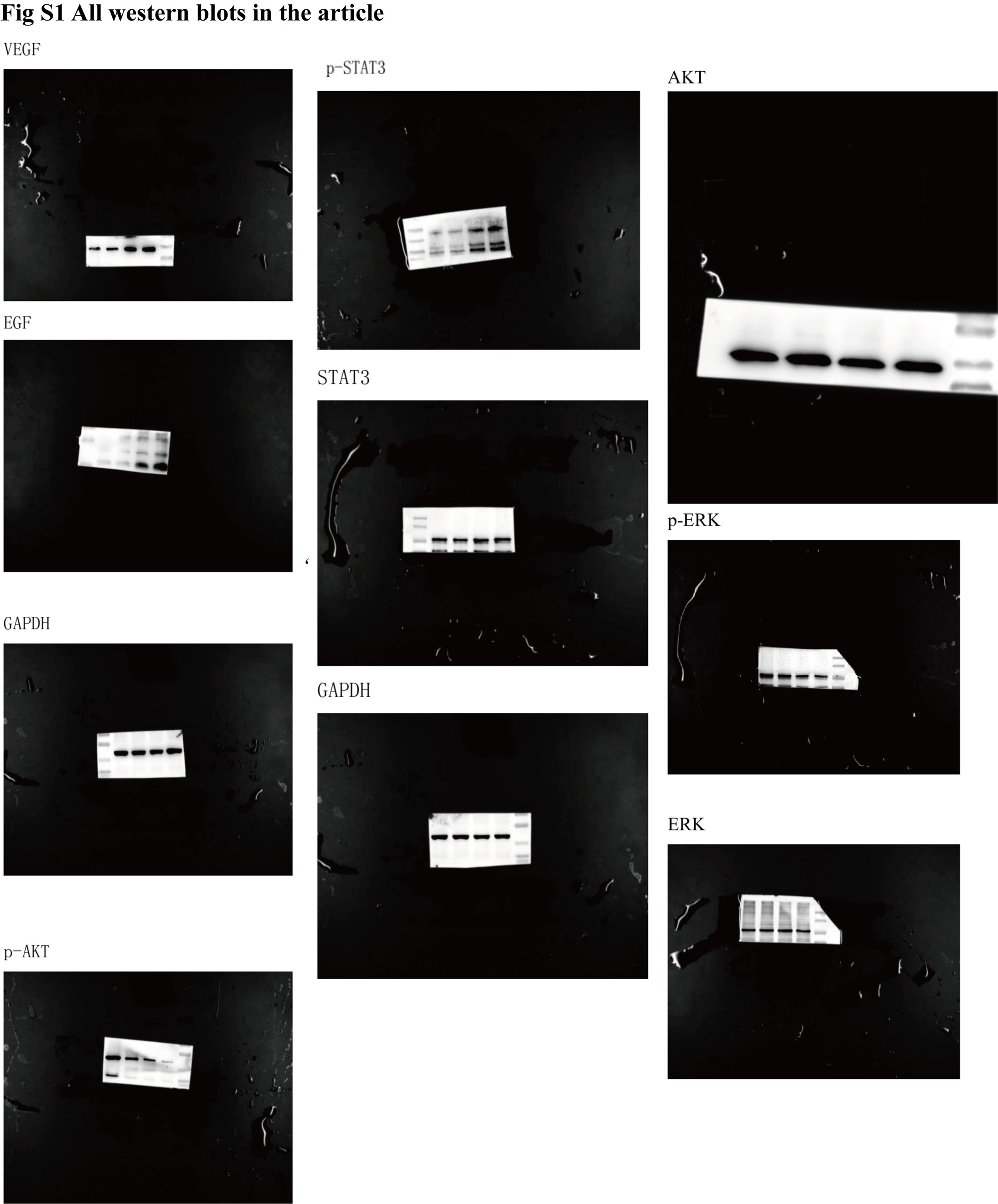

Supplement: S1 Fig — The original images of western blots. (TIF) [file pone.0279727.s001.tif]

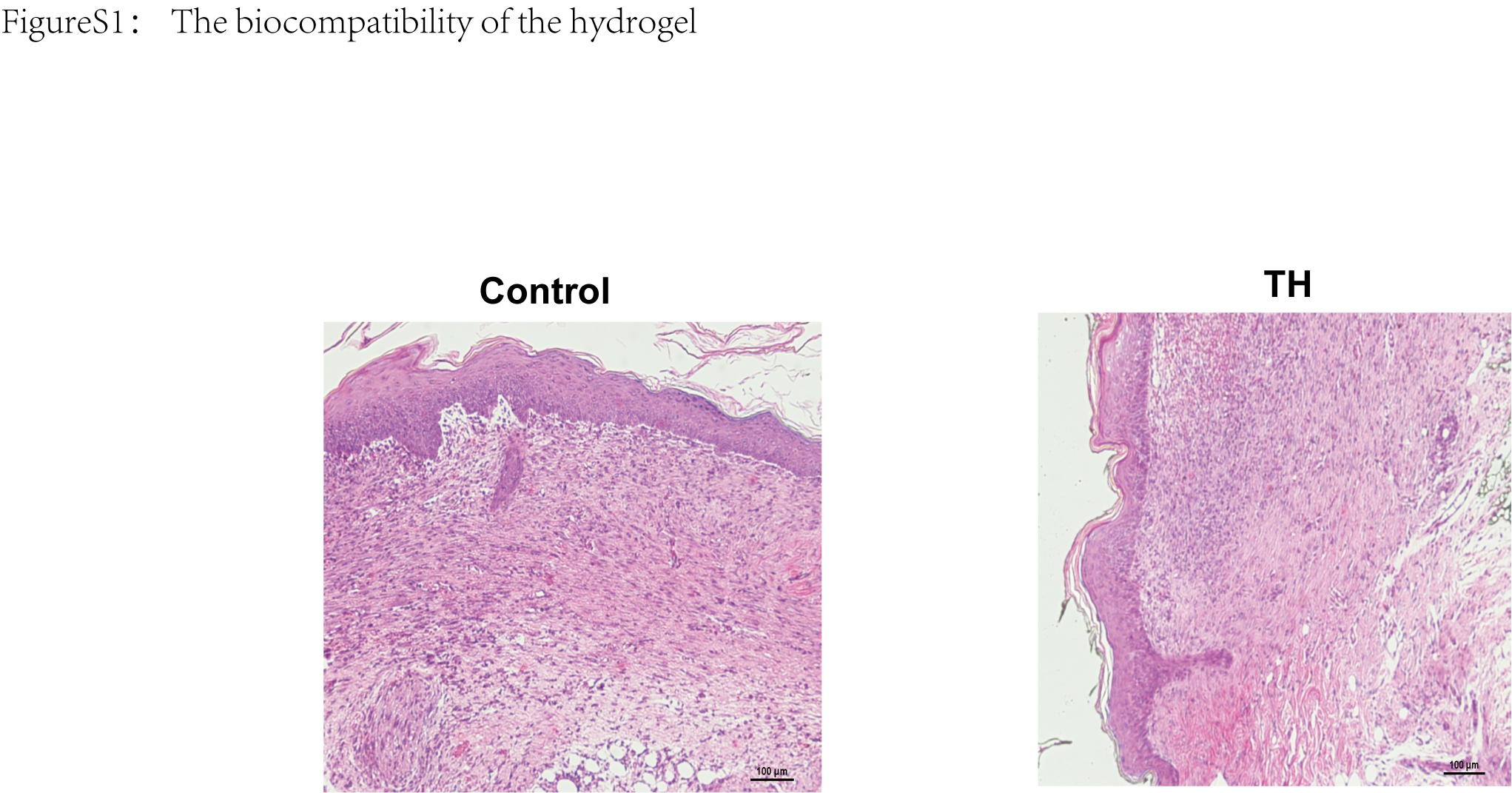

Supplement: S2 Fig — The comparison of wound skin stained with HE after using hydrogel. (TIF) [file pone.0279727.s002.tif]
