# Supplementary material for: Smart thermosensitive poloxamer hydrogels loaded with Nr-CWs for the treatment of diabetic wounds
Source: PLoS One. 2022 Dec 30;17(12):e0279727. doi: 10.1371/journal.pone.0279727 (PMC9803202; doi:10.1371/journal.pone.0279727)
Supplement: Supplementary file 2 [file pone.0279727.s003.docx]

Certificate of English Editing

To whom it may concern:

This memo certifies that one of our clients has contracted our academic editing service for the following file.

Order Number:
P-202209050535oft
Word Count:
4591 words
Date of the review:
09/05/22 (MM/DD/YY)

The English review was conducted using a two-stage process, in which a junior editor first reviewed the file, and then a senior editor conducted a final and more thorough review. All of our editors are native English-speaking professionals.

Documents receiving this certification should be English-ready for publication; however, the author has the ability to accept or reject our suggestions and changes.

We would like to emphasize that our service targets grammar and language edits. We do not rewrite the documents from scratch. If you are dissatisfied with specific revisions, please contact service@essaystar.com.

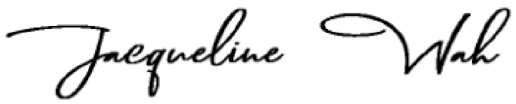

Essaystar Group

+1-208-975-4235
EssayStar, 93 S Jackson St, Seattle, WA 98104
