# Supplementary material for: Smart thermosensitive poloxamer hydrogels loaded with Nr-CWs for the treatment of diabetic wounds
Source: PLoS One. 2022 Dec 30;17(12):e0279727. doi: 10.1371/journal.pone.0279727 (PMC9803202; doi:10.1371/journal.pone.0279727)
Supplement: Attachment — Submitted filename: Response to reivewers 1.docx [file pone.0279727.s004.docx]

Dear Editors and Reviewers:

Thanks for your letter and for the reviewers' comments concerning our manuscript entitled “Smart thermosensitive poloxamer hydrogels loaded with Nr-CWs for the treatment of diabetic wounds”. Those comments are valuable for revising and improving our paper with important guiding significance. We have made correction according to the comments, revised portion are highlighted in the paper. The responds to the reviewers' comments are as follows:

Reviewer 1：

*Reviewer #1: In method section regarding release profile of Nr-CWS from hydrogel in vivo, authors still did not clarify the chemistry used for the cy5 conjugation. Is this NHS chemistry? Please add this information into your method beyond 'covalent'. How is the free dye removed and validated? This is critical information in deciding the validity of all of the drug release data. In addition, how does Cy5 disrupts this protein treatment if the modification is covalent? Another way of asking this question would be, is the Nr-CWS hydrogel treatment even more effective without Cy5 labeling?*

Response：We are very sorry that we did not add the detailed explanation to the experimental part. As you said, this reaction was made by NHS chemistry. We used the cy5 marker only to measure its release, not to add cy5 to the drug. We finally tested the absence of excess cy5 fluorescent dye in the filtrate through dialysis bags with 8000 molecular weight. Indicating that no excess fluorescent dye affects the final result. We place this revision at: （Line 171-174）.

Reviewer #3: (No Response)

*Reviewer #4: In this manuscript, the author reported hydrogel loading Nocardia rubra cell wall skeleton (Nr-CWs) to accelerates wound healing. This hydrogel would reduce inflammation, increase angiogenesis and wound healing. In general, this work is well designed and carried out. However, there also have some minor issues need to be revised:*

Response:Thank you very much for your affirmation of our research group. The members of our research group have carefully revised the questions you raised and believe that your guidance has played a decisive role in the improvement of our article. After careful research, we will respond to your questions one by one. Hope to get your approval.

1. *Why did the author choose P407 and P188 as the composition of hydrogels? The degradation of hydrogels in vivo needs to be confirmed.*

Response：Neither P407 nor P188 can reach the human body temperature and just become gel state, so the transition temperature can be controlled through matching. As for the first confirmation, we explained it from the following three aspects: First, we reviewed the literature and found that either P188 or P407 alone would not have an impact on organisms (1, 2). Secondly, we use it on the surface of the wound surface. It is more difficult for large molecules to enter the body and participate in the circulation than small molecules. Finally, we also injected the mice subcutaneously. After dissolution, we sliced the skin of the mice and observed that no other discomfort was found, so we chose the mixed material to treat the wound. This part of the supplement is modified at:(Line73-76)

2. *Please confirm that Nr-CWS was successfully loaded inside the hydrogel*.

Response：Sorry we didn't explain that clearly. First of all, we conducted comprehensive detection of the blank gel and the gel coated with the drug in Fig1. We found that the drug Nr-CWS had been encapsulated in the pores by electron microscopy, which was also verified by subsequent release experiments. Therefore, we believe that Nr-CWS was successfully incorporated into the gel. The location is marked (Line 246-247)

3. *The biocompatibility of the hydrogel needs to be confirmed before in vivo animal experiments.*

Response：As mentioned earlier, we have studied biocompatibility from three aspects. Firstly, we read the literature and found that both P407 and P188 alone have been demonstrated in new aspects of biocompatibility, which will not affect normal cell tissues (3, 4). Secondly, we tried to find the potential danger by subcutaneous injection of mice, but we did not find that these macromolecules would affect normal tissues by observing the HE staining of skin wounds (FigS2). Thirdly, we believe that when gel with drugs is applied to the wound surface, macromolecular gel is more difficult to enter the body than small molecular substances, so the impact will be smaller. And after 12h, we will clean the residual gel. From the above point of view, we believe that its biocompatibility has been well certified. This revision is placed on Line: (Line73-76)

*4.Diabetic wound healing is a complex and continuous process. The manuscript indicates that Nr-CWS in the hydrogel releases about 60% of the original within 12 h. This does not seem to meet the need for sustained wound repair. Once the release of Nr-CWS in the hydrogel is terminated, how the hydrogel promotes wound repair?*

Response：We apologize for the misunderstanding caused by our rough explanation. As is known to all, in the treatment of diabetic wounds, the wound microenvironment is very poor. In the long treatment cycle, we must carry out debridement regularly to ensure a clean and tidy wound. Therefore, we found that a clinical cycle of 12h is the most effective treatment plan, and a 12h wound cleaning has a good effect on both the patient and the wound healing. Therefore, this gel is also released once for 12h, during which the gel will not only release the drug but also provide good protection to the wound. However, the delivery environment of diabetic wound and gel in the body is different. Diabetic wound will not only have a lot of exudates, but also contact with outside air will cause harmful substances to affect wound healing. Therefore, this gel can absorb exudates and physically block harmful substances from the outside to accelerate wound healing and wound repair.We put the explanation in (Line365-370) of the article.

5. *Whether collagen deposition at the wound site after hydrogel treatment causes excessive fibrosis of the wound, explain them.*

Response:The question you raised is also the problem we met in our original study. Whether collagen fibers are hyperplasia is mainly to observe whether the arrangement of collagen fibers is disordered. When the staining is dark and the arrangement of collagen fibers is chaotic, we will consider this part as collagen fiber hyperplasia. Through the experiment, we can find that the growth and arrangement of collagen fibers are very regular after the use of gel, so we can conclude that there is no problem of excessive fiber hyperplasia.

6. *Why did the author not continue the drug release test for Nr-CWS release performance?*

Response:Thank you very much for your question, as pointed out above. Due to the particularity of the diabetic environment, wound cleaning is required regularly to ensure the best effect. However, our drug use time is just once 12h administration frequency, so we did not continue to evaluate the release performance of the drug.

7. *In the manuscript, the experimental results are described excessively, and the discussion of scientific problems is lacking. The author had better analyze the scientific problems in the manuscript in detail. In addition, the novelty of this work still needs to be highlighted in the introduction and conclusion.*

Response:Our team is very sorry for the confusion in the reading. Here, our team has carefully deleted and supplemented these parts, hoping to get your approval.Lin97-103 and Line384-388 and Line 416-426.

*Reviewer #5: In manuscript ‘Smart Thermosensitive Poloxamer Hydrogels Loaded with Nr-CWs for the Treatment of Diabetic Wounds’, the authors designed a P188/P407 poloxamers hydrogel mixed with Nr-CWS to promote angiogenesis and wound repair. This paper will be of interest in readers working in the field of Full-thickness skin defect repair. Some revisions are required to make it appropriate for publication, and the issues related to these revisions are listing below:*

Response:Thank you very much for your recognition of our team's work. The whole team is very encouraged to receive your comments. We believe that there is still a long way to go in the treatment of diabetes wounds, but we hope that our work can give some guidance to future generations. Hope to solve the problem of diabetic wound healing as soon as possible.

*Q1. There are not enough data to explain the characteristics and innovations of this study.*

Response:We are very sorry that we did not emphasize this point in the course of the article writing and the experiment. We will write the introduction and discussion section again. I have focused on replying to your question. We hope that your approval of our supplementary deletion can be obtained, and this article will be published in plos one.Lin97-103 and Line384-388 and Line 416-426.

*Q2 In ‘The slow release prolongs the duration of the drug's effect on the wound in vitro and in vivo’ why did only test the drug’s release in 12 hours? More time points should be tested.*

Response:We have this problem in our research. The most true state is that complex diabetic wounds are associated with exudation and external physical stimulation on a daily basis, resulting in slow healing problems. However, no matter which preparation of material is applied on the limited wound surface, it is impossible to completely solve these problems. So dressing change debridement has become a problem to be solved at this stage. In the long process of wound healing. Wounds cannot be solved with a single application. So we noticed this in the clinical transformation process and came up with this gel.

*Q3. We found no description of the degradation performance of poloxamers hydrogels.*

Response:The degradation performance of this gel is beyond doubt, on the one hand, single P188 and P407 have been proven (1,2). Secondly, it is difficult for macromolecular gels to enter the body from skin wounds, and we have proved in cell experiments that they do not have toxic effects on cells. Therefore, we believe that this gel is safe and reliable. In the use cycle once every 12h. The main function of our macromolecular gel is still wound protection and drug release.we did not find that these macromolecules would affect normal tissues by observing the HE staining of skin wounds (FigS2). So it's almost never metabolized in the body and it doesn't affect safety.I'm sorry, we have explained this problem. Put the modified part in Line (Line73-76)

*Q4. In ‘Fig.4D’, we didn’t see the scale bar in both 6 and 14 days*

Response:I am very sorry that we ignored this problem in our previous work. We have revised it and uploaded it again.

*Q5. In ‘Fig.4F’, we can hardly see the difference in collagen deposition between four groups in 14 days.*

Response:We can tell by the color and arrangement of the collagen fibers. We found that the structure arrangement of Nr-CWS gel was more orderly than that of blank gel. That means it heals better. However, the color of diabetic wounds was significantly lighter than that of blank gel, indicating that the density and growth of collagen fibers were far lower than that of gel group. We believe that the repair of collagen fibers is quite obvious in this comparison.

*Q6. There were still format problems. For example, the title ‘Western blot analysis’ should be bold. ‘Establishment of Diabetic diabetic wound model’ should be deleted ‘Diabetic’; the ‘℃’ in line 106 and 159 were different and we didn’t see a space between ‘0.5h,shaken’ in line 160 so on.*

Response:I'm really sorry that we have rearranged and modified all the parts.We make all ℃ in the text the same See in Line 116, 117, 147 and 171 and so on.

Reference

1.	Tan MSA, Pandey P, Lohman RJ, Falconer JR, Siskind DJ, Parekh HS. Fabrication and Characterization of Clozapine Nanoemulsion Sol-Gel for Intranasal Administration. Mol Pharm. 2022;19(11):4055-66.

2.	Kjar A, Wadsworth I, Vargis E, Britt DW. Poloxamer 188 - quercetin formulations amplify in vitro ganciclovir antiviral activity against cytomegalovirus. Antiviral Res. 2022;204:105362.

3.	Moiseev RV, Steele F, Khutoryanskiy VV. Polyaphron Formulations Stabilised with Different Water-Soluble Polymers for Ocular Drug Delivery. Pharmaceutics. 2022;14(5).

4.	Kamel R, El-Wakil NA, Elkasabgy NA. Calcium-Enriched Nanofibrillated Cellulose/Poloxamer in-situ Forming Hydrogel Scaffolds as a Controlled Delivery System of Raloxifene HCl for Bone Engineering. Int J Nanomedicine. 2021;16:6807-24.
